# Driving and Alzheimer's disease: A neuropsychological screening battery for the elderly

**DOI:** 10.1590/1980-57642018dn13-030008

**Published:** 2019

**Authors:** Lucía Crivelli, María Julieta Russo, Mauricio Franco Farez, Mariana Bonetto, Cecilia Prado, Ismael Luis Calandri, Jorge Campos, Gabriela Cohen, Patricio Chrem Méndez, Liliana Raquel Sabe, Ricardo Francisco Allegri

**Affiliations:** 1 FLENI Departamento de Neurología Buenos Aires Argentina Departamento de Neurología, FLENI, Buenos Aires, Argentina.; 2 FLENI Centro de Bioestadística, Epidemiología y Salud Pública Buenos Aires Argentina Centro de Bioestadística, Epidemiología y Salud Pública (CEBES), FLENI, Buenos Aires, Argentina.; 3 Universidad de la Costa Colombia Universidad de la Costa (CUC), Colombia.

**Keywords:** automobile driving, cognition, Alzheimer's disease, dementia, condução de veículo, cognição, doença de Alzheimer, demência

## Abstract

As life expectancy increases, there is a marked increase in the elderly population eager to continue driving. A large proportion of these elderly drive safely, however, patients with mild dementia are high-risk drivers.

**Objective:**

to identify the cognitive tests that best predict driving ability in subjects with mild dementia.

**Methods:**

28 drivers with mild dementia and 28 healthy elderly subjects underwent an extensive cognitive assessment (NACC Uniform Data Set Neuropsychological Battery), completed an adapted On Road Driving Test (ORDT) and a Driving Simulator assessment.

**Results:**

drivers with mild dementia made more mistakes on the ORDT and had slower responses in the simulator tasks. Cognitive tests correlated strongly with on road and simulator driving performance. Age, the Digit Symbol Modalities Test and Boston Naming Test scores were the variables that best predicted performance on the ORDT and were included in a logistic regression model.

**Conclusion:**

the strong correlation between driving performance and performance on specific cognitive tests supports the importance of cognitive assessment as a useful tool for deciding whether patients with mild dementia can drive safely. The algorithm including these three variables could be used as a screening tool for the detection of unsafe driving in elderly subjects with cognitive decline.

## RESUMO

À medida que aumenta a expectativa de vida, há um crescimento notável da população idosa ansiosa por continuar dirigindo. Uma grande proporção deles dirige com segurança, mas, pacientes com demência leve são condutores de alto risco.

**Objetivo:**

identificar os testes cognitivos que melhor predizem a capacidade de dirigir em indivíduos com demência leve.

**Métodos:**

28 motoristas com demência leve e 28 idosos saudáveis foram submetidos a uma extensa avaliação cognitiva (Bateria Neuropsicológica de Conjunto de Dados Uniformes NACC), completaram um teste de condução real adaptado (TCRA) e uma avaliação do Simulador de Condução.

**Resultados:**

motoristas com demência leve cometeram mais erros no TCRA e tiveram respostas mais lentas nas tarefas do simulador. Os testes cognitivos correlacionaram-se fortemente com a condução na estrada e no simulador. A idade, o Teste de Modalidades do Símbolo Digit e o Teste de Nomeação de Boston foram as variáveis que melhor predisseram o desempenho no ORDT e foram incluídos em um modelo de regressão logística.

**Conclusão:**

a forte correlação entre o desempenho na direção e os testes cognitivos específicos apoia a importância da avaliação cognitiva como uma ferramenta útil para decidir se os pacientes com demência leve podem dirigir com segurança. O algoritmo que inclui essas três variáveis poderia ser usado como uma ferramenta de triagem para a detecção de condução de risco em idosos com declínio cognitivo.

Driving competency in older adults is a necessary concern for public safety. In Argentina, 23.3% of individuals aged between 70 and 79 years suffer from cognitive impairment (CI) and this percentage increases to 42.5% in subjects older than 79 years.^1^ Currently, there is a growing number of people with these pathologies who annually renew their driver's license and this tendency is expected to grow as the population ages. Thus, public policy decisions on screening older adults, particularly those with cognitive decline, need to be comprehensively addressed.

According to a review conducted by the American Academy of Neurology (AAN), patients with mild dementia are considered to be high-risk drivers.^2^ However, the automobile is important as a means of transportation for older adults and the ability to continue driving is a key element for their independence.^3^ As driving cessation impacts directly on well-being and lifestyle,^4^ individual autonomy must be balanced with public safety.

The AAN proposed clinical guidelines with multiple tools to assess fitness to drive in elderly subjects and in patients with cognitive decline.^5^^,^^6^ Of these tests, the On-Road Driving Test (ORDT) was the only evaluation that, when used alone, proved able to determine aptitude to drive.^7^^-^9 It is considered the gold standard for determining driving competency and has been identified as the best predictor of driving ability for this age group.

The ORDT has not been implemented in Argentina by local authorities, mainly for practical and economic reasons. Identifying the neuropsychological tests that best predict actual driving performance would contribute to the design and implementation of a standardized cognitive assessment protocol for elderly people.

International studies have correlated road driving performance and simulator performance to neuropsychological measures. Paper and pencil tests and computerized programs are good predictors of unsafe driving.^10^ A meta-analysis of research examining the cognitive predictors of driving ability in older drivers identified a variety of computerized and paper and pencil tests with strong predictive value.^11^ The study emphasizes the importance of defining the gold standard for the measurement of driving ability as the ORDT or the driving simulator. In Argentina, two investigations have studied the capacity of neuropsychological tests to detect CI in elderly subjects who continue to drive automobiles. However, none of these investigations have evaluated actual driving skills prospectively, but assessed neuropsychological performance and asked questions about either driving license status or the number of motor vehicle crashes and abnormal driving behaviours using a semi-structured interview.^12^^,^^13^ The objective of the present study is to identify the cognitive tests that best predict driving ability to produce a driving assessment protocol and finally, to implement these tests in elderly adults with mild dementia.

## METHODS

### Subjects

Participants for the driving study were recruited from the Memory and Ageing Center at the Fundación para la Lucha contra las Enfermedades Neurológicas de la Infancia (FLENI), Buenos Aires, Argentina. All participants had a valid driver's license, had completed a clinical assessment, neuroimaging and neuropsychological evaluation and were screened for visual acuity or field deficits, history of alcohol or drug abuse, head trauma, major psychiatric disorder or other neurological disorders.

Two groups were included in this study: patients with mild dementia and normal controls (NC). Twenty-eight mild AD patients complying with the Alzheimer's Disease Research Centers (ADRC) inclusion and exclusion criteria for dementia of the Alzheimer's type (DAT), consistent with the criteria of the National Institute of Neurological and Communicative Disorders and Stroke and the Alzheimer's Disease and Related Disorders Association,^14^ were recruited Severity of dementia was staged as mild according to the Washington University CDR scale (CDR=1).^15^ Twenty-eight healthy subjects matched for age, gender, and educational level, recruited from a local volunteer group including patients' spouses, served as NC.

The local ethics committee approved the protocol and all subjects signed an informed consent form.

### Evaluation

Patients and controls underwent an extensive cognitive and driving evaluation at the neurorehabilitation facility of our center located in Escobar. The total duration of the work up was around 4 hours.

### Cognitive assessment

Subjects were evaluated using the official Spanish version of the Uniform Data Set (UDS) of the National Alzheimer's Coordinating Center (NACC).^16^^,^^17^ This neuropsychological battery assesses the cognitive domains most frequently impaired in dementia and consists of the following measures:

1. Orientation and cognitive screening: Mini-Mental State Examination (MMSE).^18^
2. Verbal episodic memory: Logical Memory Immediate and Logical Memory Delayed.^19^
3. Attention: Digit Span Forward and Backward, the Trail-Making Test Part A (TMT-A) and Digit Symbol Modalities Test (DSMT).^20^
4. Semantic memory and language: Category Fluency (animals and vegetables),^21^ and the Boston Naming Test (BNT).^22^
5. Executive function: Trail-Making Test Part B (TMT-B).^23^

The UDS battery was complemented by additional neuropsychological tests with proven capacity to differentiate normal aging from MCI and dementia: the Rey Auditory Verbal Learning Test (RAVLT),^24^ Rey-Osterrieth Complex Figure Test (ROCF), the Clock Drawing Test (CDT),^25^ phonological verbal fluency and Frontal Assessment Battery (FAB).^26^ Questionnaires for caregivers were also included: the Neuropsychiatric Inventory-Questionnaire (NPI-Q),^27^ the Functional Activities Questionnaire (FAQ)^28^ and the Forgetfulness Detection Scale (FDS). The NPI-Q provides a brief assessment of neuropsychiatric symptomatology. It is validated via an informant-based interview that assesses neuropsychiatric symptoms. The FAQ serves as a screening tool for evaluating instrumental activities of daily living and has proven efficacy in discriminating normal ageing, mild cognitive impairment and AD. The FDS is a modified version of the AD8 questionnaire^29^ and consists of 20 questions on the most frequently affected cognitive domains.

In the next step, subjects and caregivers completed the driving ability questionnaire suggested by the AAN which addresses features with different levels of relevance to driving competency, and includes a selection of items from the Manchester Driver Behavior Questionnaire. As stated by the AAN, the main purpose of the questionnaire is to qualitatively determine the driving risk in elderly patients and in subjects with dementia, and has not been validated for quantitative determination of driving risk. Nonetheless, we included the result of its total score in our analysis. Subjects completed the patient version of the questionnaire and caregivers completed the informant version.

### Driving assessment

Driving assessment consisted of a driving simulator evaluation and a road driving test.

***1) Simulator.*** The Doron L300 simulator was used for this assessment. We selected two different subtests to be analyzed:
  • 1.a.Traffic Signal Reaction Task: In this task subjects must identify traffic signals on the screen and use the steering wheel or the brake to respond appropriately as soon as they see a valid signal. Accuracy of responses are computed.
  • 1.b. Brake Reaction Task: This is a reaction time test. One or two red lights are displayed on the simulator screen in random order. Subjects must press the brake as soon as they see two red lights simultaneously. Reaction to the target is measured in distance (feet driven from target presentation until response).
***2. Driving Assessment:*** This test has two parts:
  • 2a) ORDT. We performed this test on the inner streets of the hospital premises. The subject has to drive their own vehicle accompanied by an evaluator. The road has a total length of 3 kilometers and includes intersections with traffic lights, a steep slope, a pedestrian crossing, traffic signs and a parking area (to test parking at 90 and 45 degrees and parallel parking). The course is simple, but the car is exposed to ordinary traffic. General behavior, compliance with traffic signals, ability to complete the itinerary and capacity to follow instructor's directions were assessed. The instructor measured driving ability using a standardized protocol with 31 variables (see Table 2).
  • 2b) Traffic Signal Recognition Task: subjects had to recognize 10 traffic signals presented on cards.

### Statistical analysis

Demographic and clinical characteristics of the subjects were summarized with the use of descriptive statistics, and between-group differences were assessed by means of the *t*-test or Mann-Whitney test. The non-parametrically distributed data were expressed as median values with interquartile ranges and analyzed using the Kruskal-Wallis test for between-group comparisons, as well as the Mann-Whitney test for comparisons between pairs of groups when appropriate.

A number of scores were developed to analyze the relationship of the neuropsychological tests with the Driving Assessment. The Driving Score is the sum of correct responses on the driving test (0-31); The Signal Recognition Score is the total amount of correctly identified traffic signals (presented on cards); The Traffic Signal Reaction Score is the percentage of correctly identified signals in the simulator (0-100%), and finally the Brake Reaction Score, which measures reaction distance (measured in feet).

A univariate logistic regression model was used to calculate correlation coefficients between driving and cognitive variables. Furthermore, a multivariable logistic regression model was built in a forward fashion including all variables significant at the 0.15 cut-off point in univariate analysis. Mutually adjusted odds ratios (OR) for driving ability and corresponding β coefficients were calculated. Model calibration was assessed by comparing predicted and actual patients with driving score and the goodness of fit was estimated by the Hosmer-Lemeshow χ^2^ test. Model discrimination was evaluated with the C-statistic. A simple scoring system was developed and points were assigned to each variable based on the magnitude of its regression coefficient. A total driving risk score was then calculated for each individual as the sum of points for each variable. A *p*-value ≤0.05, was considered statistically significant and all tests were two-tailed. Stata version 12.1 was used for all analysis.

## RESULTS

### Demographic and cognitive results

Demographic characteristics of the study sample are presented in Table 1. Groups did not differ in gender composition, age or years of education.

**Table 1:** Demographic and cognitive characteristics of the study sample.

		NC (n=28)	Patients (n=28)	p
**Demographic variables**	Age	73.7±4.9	76±4.7	ns
	Gender (F:M)	12:16	6:22	ns
	Education (years)	14.7±3.3	12.9±4.4	ns
**Cognitive assessment**	MMSE	29 (28-30)	26 (20-30)	<0.0001
	Clock Drawing Test (mean, range)	3 (2-3)	3 (1-3)	ns
	Logical Memory Immediate Recall	24.5±6.6	10.3±6.4	<0.0001
	Logical Memory Delayed Recall	19.4±7.3	4.17±6.6	<0.0001
	Logical Memory Recognition Trial	16.25±2.3	7.1±5.1	<0.0001
	Digit Span Forwards	6.14±1	5.64±0.9	ns
	Digit Span Backwards	4.3±1	3.7±0.7	<0.001
	Semantic Verbal Fluency (animals)	20.8±6.2	13±4.9	<0.0001
	Semantic Verbal Fluency (vegetable)	13.7±3.9	8.1±3.7	<0.0001
	Phonologic verbal Fluency	15.8±4.6	11.3±4.2	<0.001
	TMT-A	36 (24-94)	57.5 (26-123)	<0.001
	TMT-B	81.5 (52-193)	161 (44-335)	<0.0001
	DSMT	39.5±11.6	26.75±10.3	<0.0001
	BNT (mean, range)	28.5 (20-30)	24.5 (9-30)	<0.0001
	RAVLT (trials 1-5)	37.2±8.5	21.7±8.8	<0.0001
	RAVLT Delayed Recall	7.1±3.2	1±2.5	<0.0001
	RAVLT Recognition trial	11.4±2.3	6.75±5.1	0.0001
	RAVLT intrusions (mean, range)	1.5 (0-8)	2 (0-13)	ns
	RAVLT false positives	1 (0-4)	1.5 (0-14)	ns
	ROCFT Copy	33.6±3.2	31.2±3.9	<0.01
	ROCFT Delayed Recall	15.7±6.9	3.9±5	<0.0001
	FAB (0-18)	16.2±1.2	14.4±2.1	<0.001
	GDS	2.2±1.9	2.5±2.4	ns
	NPIQ	1.25±1.7	4.5±2.9	<0.0001
	FAQ	1.7±4.3	7.2±6	<0.001
	FDS	1 (0-12)	8.5 (1-18)	<0.0001
	AANDQ Patient	19.3±4.6	19.3±5.9	ns
	AANDQ Caregiver	21 (10-34)	25 (14-41)	ns

*Values expressed as mean±standard deviation with the exception of the Clock Drawing Test, the BNT, the intrusions of the RAVLT and the FDS. ns: not significant. TMT: Trail-Making Test; DSMT: Digit Symbol Modalities Test; BNT: Boston Naming Test; RAVLT: Rey Auditory Verbal Learning Test; ROCFT: Rey-Osterrieth Complex Figure Test; FAB: Frontal Assessment Battery (FAB); GDS: Geriatric Dementia Scale; NPI-Q: Neuropsychiatric Inventory; FAQ: Functional Assessment Questionnaire, FDS: Forgetfulness Detection Scale; AANQ: American Academy of Neurology Driving Questionnaire.

As expected, patients had a poorer performance on several neuropsychological tests, revealing mild dementia deficits, compared to controls (Table 1). These tests comprised the MMSE, Memory tests (Logical Memory, RAVLT, Delayed Recall ROCF), language tests (Verbal Fluency, BNT), visuospatial abilities (ROCF), executive functions (FAB; TMT-B, Digit Span Backwards) and attention (DSMT, TMT-A, Digit Span Forwards). Patients differed significantly compared to NC on the questionnaires administered to informants (NPIQ, FAQ and FDS).

However, the analysis of the results of the driving questionnaire revealed no differences between NC and dementia patients on either form of the driving ability questionnaire (self-reported questionnaire and family-reported questionnaire).

### Driving results

As shown in Table 2, patients made more mistakes than NC on the ORDT and consequently had significantly lower Driving Scores (p<0.0001). In this test, the variables that had greatest impact were: the ability to recognize turning direction, planning before turning, following traffic signals, driving carefully, understanding verbal commands, responding correctly to verbal commands and learning new orders.

**Table 2:** Driving assessment in patient and control group.

	NC (n=28)	Patients (n=28)	p
On Road Driving Test*			
1. Remains controlled in the transit lane.	96.3	60.7	<0.01
2. Correctly determines the circulation lane	81.5	46.4	<0.01
3. Recognizes the direction to turn	93	36	<0.0001
4. Signals properly	44.4	32.1	ns
5. Turning Speed.	89	68	ns
6. Recovery after turning.	92.6	67.9	<0.05
7. Positioning of the vehicle before and after turning.	96.3	67.9	<0.05
8. Signals adequately.	44.4	39.3	ns
9. Look through the mirrors.	100	89.3	ns
10. Turns head to look.	88.9	92.9	ns
11. Is able to accelerate gently	92.6	78.6	ns
12. Is able to adjust or modify the speed according to the transit or the condition of the route.	81.5	50	<0.05
13. Is able to maintain a constant speed.	92.6	67.9	<0.05
14. Is able to stop gently.	96.3	75	<0.05
15. It is capable of maintaining strong, consistent braking.	100	75	<0.05
16. Capable of curbing in appropriate place.	100	75	<0.05
17. Follows and respects the rules of the road.	88.9	35.7	<0.0001
18. Thinks and plans in advance before turning.	96.3	32.1	<0.0001
19. Responds appropriately to any dangerous situations.	96.3	64.3	<0.01
20. Show courtesy to interact with other drivers.	100	79	<0.05
21. Drives carefully.	100	50	<0.0001
22. Maintains enough space between his car and others.	100	85.7	ns
23. Adequately responds to transit control mechanisms.	92.3	48.1	0.001
24. Is able to park at 45º.	77.8	92.9	ns
25. Is able to parallel park.	51.9	35.7	ns
26. Is able to park at 90º.	63	42.9	ns
27. Responds correctly to verbal instructions.	100	35.7	<0.0001
28. Understands verbal instructions.	92.6	25	<0.0001
29. Is able to learn and obey new orders.	88.9	28.6	<0.0001
30. Can enter the vehicle without help.	100	92.9	ns
31. Can exit the vehicle without help.	100	92.9	Ns
Traffic Signal Recognition Task*			
No Entry	74.1	32	<0.01
No U-turn	100	78.6	<0.05
No overtaking	92.6	67.9	<0.05
First Aid Post	100	89.3	ns
Curve	100	100	ns
Narrow bridge	88.9	64.3	ns
No parking	96	85.7	ns
Crossroads	96.3	85.7	ns
Narrow road ahead	85.2	57	<0.05
Pedestrian Crossing	92.6	57.1	<0.01
Driving Assessment-Global Scores**			
ORDT Score	28.9±4.7	18.5±7.1	<0.0001
Traffic Signal Recognition Task score (tot signals, correctly identified, median range)	10 (7-10)	7.5 (2-10)	<0.0001
Simulator Brake Reaction score (feet)	48.5±7	57.3±16.8	<0.01
Simulator Traffic Signal reaction task score (% correct responses)	83%	49%	<0.0001

* Values are expressed in percentage of correct answers.

** Values are expressed as mean±standard deviation with the exception of the Traffic Signal Recognition Task score and the Simulator Traffic Signal reaction task score.

Differences favoring NC were also present on the Traffic Signal Recognition Score (p<0.0001). The signals that generated most differences were “No entry for vehicles”, “U-turn prohibited”, “no overtaking”, “narrow road” and “pedestrian crossing”.

Analysis of performance in the simulator revealed that the patient group had significantly slower responses on both tasks, as shown by the Traffic Signal Reaction Score (p<0.0001) and the Brake Reaction Score (p<0.01).

### Correlation analyses

Using regression analyses as described above, the TMT-B, Verbal semantic Fluency and FDS were identified as important predictors of driving performance. These tests correlated significantly with all the driving scores (see Table 3).

**Table 3:** Cognitive predictors of driving skills. Correlation between Driving Assessment and Neuropsychological Assessment.

	Driving assessment			Simulator tasks	
	ORDT	Signal recognition		Brake reaction time task	Traffic signal reaction task
Age	0.05	n.s.		<0.05	n.s.
Gender (F:M)	n.s	n.s.		n.s.	n.s.
Education (years)	n.s	n.s.		n.s.	n.s.
MMSE	<0.05	<0.001		n.s.	<0.001
Clock Drawing Test (mean, range)	n.s.	<0.01		n.s.	<0.01
Logical Memory Immediate Recall	<0.001	<0.001		n.s.	<0.001
Logical Memory Delayed Recall	<0.001	<0.01		n.s.	<0.001
Logical Memory Recognition Trial	<0.01	<0.001		n.s.	<0.001
Digit Span Forwards	n.s.	n.s.		n.s.	n.s.
Digit Span Backwards	n.s.	n.s.		n.s.	<0.01
Semantic Verbal Fluency (animals)	<0.01	<0.01		<0.05	<0.001
Semantic Verbal Fluency (vegetable)	<0.001	<0.01		n.s.	<0.001
Phonologic verbal Fluency	n.s.	n.s.		n.s.	<0.01
TMT-A	<0.01	n.s.		<0.05	<0.001
TMT-B	<0.01	<0.05		<0.01	<0.001
DSMT	<0.01	<0.01		n.s.	<0.001
BNT	<0.01	<0.001		n.s.	<0.001
RAVLT (trials 1-5)	<0.001	<0.001		n.s.	<0.001
RAVLT Delayed Recall	<0.01	<0.001		n.s.	<0.001
RAVLT Recognition trial	n.s	<0.01		n.s.	<0.001
RAVLT intrusions	n.s	n.s.		n.s.	n.s.
RAVLT false positives	<0.01	n.s.		n.s.	n.s.
ROCFT Copy	<0.001	<0.001		n.s.	<0.001
ROCFT Delayed Recall	n.s	n.s.		n.s.	n.s.
FAB (0-18)	<0.05	<0.01		n.s.	<0.001
GDS	n.s	n.s.		n.s.	n.s.
NPIQ	<0.01	<0.01		n.s.	<0.05
FAQ	<0.01	<0.05		n.s.	<0.01
FDS	<0.001	<0.01		<0.05	<0.001
AANDQ Patient	n.s	n.s		n.s.	n.s.
AANDQ Caregiver	n.s	n.s		n.s.	n.s.

*ns: not significant. TMT: Trail-Making Test; DSMT: Digit Symbol Modalities Test; BNT: Boston Naming Test; RAVLT: Rey Auditory Verbal Learning Test; ROCFT: Rey-Osterrieth Complex Figure Test; FAB: Frontal Assessment Battery (FAB); GDS: Geriatric Dementia Scale; NPI-Q: Neuropsychiatric Inventory; FAQ: Functional Assessment Questionnaire, FDS: Forgetfulness Detection Scale; AANQ: American Academy of Neurology Driving Questionnaire.

The other group of tests correlated with three of the four scores of driving ability. The TMT-A correlated with both simulator scores (Traffic Signal Reaction Task and Brake Reaction Time Task) and with the Driving Test. The other group of tests, including the MMSE, Logical Memory, Semantic Verbal Fluency (vegetables), DSMT, BNT, RAVLT, ROCFT (copy), FAB, NPI-Q and FAQ, correlated significantly with the ORDT, the Traffic Signal Recognition Task and with the Traffic Signal Reaction Task of the simulator, but not with the Brake Reaction Task.

### Logistic regression model analyses

Aptitude to continue driving was predicted using a logistic regression model (see Table 4). Age, SDMT and BNT were the variables that best predicted performance on the driving test and were included in this model. The cut-off score of age was 72 years old (72 or more scores 0 points). On the SDMT, a score of 27 points or less is attributed 0 points, a score of 28 to 38 is given 0.5 points and a score of 39 or more is attributed 1 point. The BNT variable scores a maximum of 1 point; obtaining a score below 23 is worth 0 points and a score of 23 or more is given 1 point. Each of the three variables (age, SDMT, BNT) score a maximum of 1 point. A cut-off score of 1.5 was established to determine whether subjects were fit to continue driving. A total score of 1.5 or more indicates that subjects can drive. No single variable is enough to attain the cut-off score, but a good performance on two of the three variables can reach the threshold.

**Table 4:** Comparison of performance on ORDT and performance predicted by cognitive tests selected.

		Performance prediction	
		Failed (<1.5)	Approved (≥1.5)
On Road Driving Test	Failed (<38/46)	8	3
	Approved (≥38/46)	3	14

## DISCUSSION

The goal of this study was to identify the neuropsychological tests that best predict driving performance in elderly subjects with cognitive impairment.

The Driving Assessment used in our study, includes an On Road Driving assessment and an evaluation in a driving simulator, and provides the advantage of accurately measuring the ability to react to complex situations. The neuropsychological battery also proved to be sensitive and specific for the detection of cognitive decline.

Our results showed that drivers with mild dementia had a poorer performance than NC, both on the Cognitive Assessment and on the Driving Assessment. These differences could not be explained by age, sex or educational level, as these variables were matched. These results are consistent with findings of other studies on driving and dementia.^30^^-^32

As previously shown in other studies, subjects´ self-reported driving skills were not considered a valid indicator of driving performance.^33^ In addition, and contrary to our expectations, the informants' version of the driving skills questionnaire did not differ significantly between the groups or show any correlation with the actual driving skills assessed. This result can be explained in part by the fact that this questionnaire was recommended by the AAN as a qualitative tool and not a quantitative measure as used in the present study. Bixby et al. (2015) reported similar results to the present study and in both cases the main informants who completed the questionnaire were the spouses of patients.^34^ This could be a significant limitation since a personal bias could affect spouses´ responses. Variables such as the desire to evade interpersonal conflicts, to dismiss the progression of the disease or even the useful role of the patient as a driver may account for some of the reasons for the biases in response.^35^

Results on the neuropsychological tests showed a strong correlation with actual performance in the Driving Assessment. The neuropsychological tests that correlated with all components of the Driving Assessment were the TMT-B, semantic verbal fluency (animals) and the FDS. Given these results, and considering the simplicity of the administration process, the TMT-B, FDS and semantic verbal fluency can be recommended as the optimal tools for cognitive screening.

In addition to these three tests, other cognitive tests also correlated with different driving scores. The TMT-A did not correlate with the Traffic Signal recognition Score, but correlated with the remaining driving scores (Driving Score, Reaction to Traffic Signal Score, Brake Reaction Score).

Another group of cognitive tests that correlated with the Driving Assessment Score, Traffic Signal Recognition Score, and the Traffic Signal Reaction Score were the MMSE, logical memory, semantic fluency (vegetables), the DSMT, BNT, RAVLT, ROCFT (copy stage only), FAB, NPI-Q and the FAQ.

This group of tests could be used as a comprehensive cognitive assessment battery when subjects fail on screening tests.

A multivariate logistic regression model was built based on ORDT performance and on neuropsychological and clinical variables. An algorithm was designed in order to identify the neuropsychological tests that best predict driving skills as measured only by the ORDT. Age, the SDMT and the BNT were included in this model, each of which accounted for an equal part of the total score (maximum one point each).

Epidemiological studies have identified age as one of the strongest predictors of dementia, thus, it is expected to behave as a predictor of cognitive impairment that can interfere with driving skills.

SDMT has been reported as a robust measure of information processing speed and attention. These functions are important components for overall successful motor programming and require coordination for adequate driving. In our study, the SDMT correlated significantly with driving performance.

BNT is a language task that taps lexical access, one of the most vulnerable areas of language in mild dementia. In this study, the BNT differed significantly between groups and correlated strongly with performance on the driving test.

The successfully designed algorithm can be used as a screening tool to detect cognitive impairment that correlates with unsafe driving in the elderly population. Administration of these tests ( BNT, SDMT) is rapid and requires only inexpensive materials.

To our knowledge, this is the first study to assess prediction of driving and cognitive skills and their correlation in elderly subjects in Argentina. Previous studies have attempted to investigate cognitive status and driving skills in elderly subjects using questionnaires, but none have conducted a prospective evaluation of cognitive and driving skills.

The sample size and high level of education are limitations of this study. Future research should enlarge and diversify the study group and produce ROC curves in order to establish proper cut-off scores for each of the tests.

Proper neuropsychological assessment can serve as an important component in Driving Assessment. Cognitive tests that correlated with the driving assessment in this study could be used as predictors of the ability to drive in older adults. They can provide information on specific cognitive difficulties that could indicate a greater chance of unsafe driving.
